# hTERT Transduction Extends the Lifespan of Primary Pediatric Low-Grade Glioma Cells While Preserving the Biological Response to NGF

**DOI:** 10.3389/pore.2021.612375

**Published:** 2021-04-02

**Authors:** Ornella Franzese, Angela M. Di Francesco, Daniela Meco, Grazia Graziani, Gabriella Cusano, Lauretta Levati, Riccardo Riccardi, Antonio Ruggiero

**Affiliations:** ^1^ Department of Systems Medicine, University of Rome Tor Vergata, Rome, Italy; ^2^ Institute of Internal Medicine, Periodic Fever and Rare Diseases Center, Fondazione Policlinico A. Gemelli, IRCCS, Rome, Italy; ^3^ UOC di Oncologia Pediatrica, “Fondazione Policlinico Universitario A. Gemelli”, IRCCS, Rome, Italy; ^4^ Molecular Oncology Laboratory, IDI-IRCCS, Rome, Italy

**Keywords:** low-grade glioma, senescence, hTERT, NGF (nerve growth factor), differentiation

## Abstract

The neurotrophin nerve growth factor (NGF) modulates the growth of human gliomas and is able to induce cell differentiation through the engagement of tropomyosin receptor kinase A (TrkA) receptor, although the role played in controlling glioma survival has proved controversial. Unfortunately, the slow growth rate of low-grade gliomas (LGG) has made it difficult to investigate NGF effects on these tumors in preclinical models. In fact, patient-derived low-grade human astrocytoma cells duplicate only a limited number of times in culture before undergoing senescence. Nevertheless, replicative senescence can be counteracted by overexpression of hTERT, the catalytic subunit of telomerase, which potentially increases the proliferative potential of human cells without inducing cancer-associated changes. We have extended, by hTERT transduction, the proliferative *in vitro* potential of a human LGG cell line derived from a pediatric pilocytic astrocytoma (PA) surgical sample. Remarkably, the hTERT-transduced LGG cells showed a behavior similar to that of the parental line in terms of biological responses to NGF treatment, including molecular events associated with induction of NGF-related differentiation. Therefore, transduction of LGG cells with hTERT can provide a valid approach to increase the *in vitro* life-span of patient-derived astrocytoma primary cultures, characterized by a finite proliferative potential.

## Introduction

Tumors of glial cell origin represent almost 70% of pediatric brain tumors, with astrocytomas accounting for 50% of all of them [1, 2], and are usually regrouped into low- [WHO grade I, pilocytic astrocytoma (PA) and WHO grade II] and high-grade tumors (WHO grade III, anaplastic astrocytoma and WHO grade IV, glioblastoma) [3]. PAs are among the most prevalent tumors of the central nervous system (CNS) in infancy, occurring either sporadically or in 15–40% of children with neurofibromatosis type 1 (NF1) [4]. PAs are usually located in the cerebellum; however, a low-grade astrocytoma subgroup involves the optic nerve and other optic structures (i.e., optic gliomas, OGs). Cerebellum PAs show a good prognosis when a complete surgical resection of the lesion is feasible, with a 5-years overall survival of more than 90% [1, 5]. Neurological complications, endocrine deficiencies or cognitive impairment can represent direct consequences of the tumor mass, due to the CNS location of these malignancies. One of the main features of OGs is their indolent or unpredictable growth rate that can produce vision loss involving both the anterior and retro-chiasmatic optic pathways long before diagnosis.

Recent studies have demonstrated that the administration of exogenous Nerve Growth Factor (NGF) as eye-drops, either in children or adults with severe visual impairment due to low-grade OGs, is associated with a significant improvement of both the visual evoked potential and the ocular function [6, 7]. NGF is a member of the neurotrophin family, with a well-established role in the development and maintenance of sensory, sympathetic and cholinergic neuronal populations [8]. NGF biological effects are mediated by the engagement of specific receptors: the tropomyosin receptor kinase A (TrkA) and the p75 NGF receptors, which display pleiotropic effects in inducing cell differentiation, regulating apoptosis and supporting neuron survival [9, 10]. NGF can induce cell differentiation through the activation of different TrkA down-stream signaling pathways, including Ras/Raf/MEK/ERK [11–13]: in particular, the role of sustained ERK activity has been proven necessary and sufficient for NGF-induced differentiation [13].

The low affinity p75 receptor, belonging to the tumor necrosis receptor family, is involved in neuronal apoptotic signals induced by the neurotrophins [14]. However, in astrocytes, although not directly involved in apoptosis induction, it exerts an inhibitory effect on cell proliferation [15]. Endogenous NGF expression has been reported to increase with glioma aggressiveness [16], while exogenous NGF is involved in the regulation of glioma cell growth *in vivo* [17]. However, the role played by NGF in tumor cell survival has been proven contradictory, ranging from inhibiting cell proliferation [18, 19] to being mitogenic [20, 21]. Based on these findings and taking into account the positive clinical outcome obtained with conjunctival NGF administration in terms of visual rescue in OG patients, the effect of exogenous NGF on low-grade glioma remains an open question.

The slow growth degree of low-grade gliomas (LGGs) has made difficult to obtain primary models for preclinical studies regarding NGF effects on these tumors. In fact, patient-derived human low-grade astrocytoma cells can divide only a finite number of times in culture before undergoing replicative senescence [22].

Cellular senescence is characterized by a complex scenario with many potential players. Oncogene-induced-senescence can be driven by mutations occurring in key-oncogenes such as KRAS, BRAF, PTEN and NF1 that, in the absence of additional cooperating mutations, may act as a mechanism of tumor suppression leading to slow growing benign lesions [23, 24]. Actually, PAs are slow growing tumors which appear genetically distinct from diffuse astrocytomas, showing low proliferation index and displaying aberrant activation of the MAPK pathway [25, 26] that may trigger senescence in PAs partly through the activation of the p16 pathway [20, 27].

Replicative senescence in human cells can be overcome by the overexpression of the catalytic subunit of telomerase hTERT, leading to the generation of stable, non-oncogenic lines of a variety of cell types [28]. Indeed, constitutive hTERT overexpression in primary cultures of different tissue origin can increase the proliferative potential of cells without causing cancer-associated changes or altering cell phenotypic features. PAs are commonly telomerase negative [29], although some heterogeneity in the expression and distribution of hTERT has been observed [30]. Since hTERT overexpression has not been directly linked to astrocytoma cell invasion [31], transduction of PA cells with the catalytic subunit of telomerase may potentially lead to an increased lifespan in these cells without altering the phenotype in terms of tumor aggressiveness. Interestingly, hTERT transduction can contribute to circumvent cellular senescence in astrocytes, without promoting a transformed phenotype [32, 33].

Herein, by hTERT transduction, we have extended the proliferative potential of a LGG cell line which has retained similar biological responses to NGF treatment compared to the parental cells. Expression of TrkA and p75 levels were not affected by hTERT transduction, neither were cell proliferation and molecular events associated with NGF-related induction of differentiation, suggesting that hTERT transduction can provide a valid tool for *in vitro* preclinical studies on patient-derived LGG cells.

## Materials and Methods

### Cell Cultures

Cell lines were obtained from PAs of different grades, developed in 3–16 years old patients. Cell lines were established *in vitro* as monolayer cultures, with doubling times between 24–48°h, and showed a mixture of stellate and bipolar morphologies, with cells presenting polygonal, cuboidal or flattened appearance. Low-grade glioma cell lines were isolated from fresh tissues collected from the surgical theater. Briefly, fresh tissues were collected in complete DMEM medium supplemented with 20% FBS and antibiotics (Sigma-Aldrich, St. Louis, MO, United States), then mechanically minced straight away and left in culture. Once cells started growing, the FBS concentration in culture medium was reduced to 10%. Cells were followed in culture for weeks, and the proliferative capacity was assessed calculating the number of population doublings (PDs), using the formula log_10_ [total no./start no.]/ml until cells proceeded to senescence [34, 35]. Of all attempts, two cell cultures (named LGG1 and LGG2) reached 11 passages in culture over a period of 5 months. Genotyping studies of these cells showed a good match compared to their corresponding original tissues, indicating that no contamination had taken place in culture. The high-grade glioma (HGG) cell lines, A172 and MG-U87, used for comparative studies on NGF effects, were purchased from ATCC® (Manassas, VA, United States).

### Chemicals and Antibodies

Human recombinant NGF was kindly provided by Dr L. Manni (Institute of Translational Pharmacology, CNR, Rome, Italy). Antibodies (Abs) against β-actin, TrkA, p75, GFAP, p‐16, used for Western Blot were from Santa Cruz Biotechnology (Dallas, TX, United States). Abs anti‐extracellular signal regulated kinase (ERK)1/2, anti‐p‐ERK1/2 (Thr202/Tyr204), anti-AKT, anti‐p‐AKT (Ser473), anti-p38 MAPK and anti-pp-38 MAPK (Thr180/Tyr182) were from Cell Signaling Technology (Danvers, MA, United States); anti-MASH-1 Ab was from Thermo-Fisher (Waltham, MA, United States). Abs for immunofluorescence studies were against: GFAP (Thermo-Fisher), vimentin (Santa Cruz Biotechnology), synaptophysin, S100 β, nestin, all from Sigma-Aldrich. Alexa Fluor 488 F (Ab)2 fragment of goat anti-rabbit IgG and Alexa Fluor 488 F (ab)2 fragment of goat anti-mouse IgG were used as secondary Abs for IF (Jackson Immunoresearch, Ltd. Europe). Eukitt from Sigma-Aldrich was employed as mounting solution.

### Virus Production and Infection

The human epithelial cell line 293T was maintained in DMEM supplemented with 10% FCS. For transfection experiments, 15 μg of the packaging constructs pCMVDR8, 2,5 μg of the VSV-G expressing plasmid pMD.G and 10 μg of the transfer plasmid pHR0-CMV-hTERT-IRES2-GFP or the pHR0-CMV-IRES2-GFP control (a kind gift from Dr A. Cara, ISS, Rome) were introduced into 293T cells using the Calcium Phosphate Method (Promega, Madison, WI, United States). Transduction was performed by overnight incubation of PA cells with viral supernatants. Between 12–16 h after infection, the virus-containing media was removed, and cells were incubated with fresh media.

### Detection of GFP Expression in Lentiviral-Transduced Cells

The expression of the reporter gene GFP was analyzed by evaluation of the integration of GFP into the target cell’s genome, as GFP expression is reduced when it is positioned as the second gene downstream of the IRES sequence, limiting its flow cytometric detection [36]. Integration of GFP into the cell genome was analyzed by polymerase Chain Reaction (PCR) amplification from purified genomic DNA. PCR analysis demonstrating GFP integration (250°bp product) was performed on control and hTERT transduced cells as described [34]. hTERT expression in transduced cells was also assessed by intracellular flow cytometry detection, following cell permeabilization with Cytofix/Cytoperm (BD, Franklin Lakes, NJ, United States) and staining with anti-hTERT Ab (Santa Cruz) and FITC goat anti-mouse IgG as secondary Ab (Thermo-Fisher).

### Wound Healing Retard Assay

For the wound healing assay, cells were grown to confluence and serum-starved for 24 h in DMEM without FBS. Monolayers were gently scraped to produce a wound area, then washed and cultured in the presence or absence of NGF (100 ng/ml). Progression of wound closing was monitored by taking phase-contrast pictures at 48 h by Canon Power shot G5 (Tokyo, Japan).

### Analysis of Senescence and Immunofluorescence Staining

β-galactosidase assay, allowing detection of β-galactosidase activity at pH 6 in senescent cells, was performed following instructions provided by the manufacturer.

For immunofluorescence studies, briefly, cells were seeded and, when indicated, treated with NGF (100 ng/ml) for 72°h, fixed in 4%, paraformaldehyde, permeabilized in 0.3% triton X-100, incubated with primary Ab overnight at 4°C or 1 h at 37°C, then with secondary Ab and stained with DAPI before mounting. Slides were stored at −30°C in the dark until acquisition.

### Western Blot Analysis

Cells were washed in cold PBS, scraped on ice and lyzed in ice-cold lysis buffer (0.5% sodium deoxycholate, 20 mM Tris–HCl, pH 7.4, 0.1 M NaCl, 1% Nonidet P40, 5 mM MgCl_2_, 1 mM DTT, with protease inhibitors) for 20 min on ice. Samples were separated by SDS-PAGE, transferred to Hybond-P membranes (GE Healthcare, Chicago, IL, Unites States), probed with specific primary and horseradish peroxidase-conjugated secondary Abs, then binding visualized by chemiluminescence (GE Healthcare) with Chemidoc XRS System (Bio-RaD, Hercules, CA, United States). Western Blot analysis of MASH-1 expression was also performed in the presence of K252a, a pan Trk inhibitor (Sigma-Aldrich), [37]. Densitometry was performed using Imager ChemiDoc XRS System.

### BRAF exon15 Sequencing

Genomic DNA was isolated from LGG1, LGG2 and LGG2-hTert cell lines using DNeasy Blood and Tissue Kit (Qiagen, Valencia, CA) following manufacturer’s protocol. Quality and concentration of the DNA samples were examined by NanoDrop ND-1000 (Thermo Fisher Scientific, Waltham, MA) and 25 ng of DNA was used to perform PCR amplification of BRAF exon15 with the following primers: 5’-CCT​AAA​CTC​TTC​ATA​ATG​CTT​GCT-3’ (forward) and 5’- GGC​CAA​AAA​TTT​AAT​CAG​TGG​A -3’ (reverse). PCR conditions were set up according to manufacturer’s instructions of Invitrogen™ Platinum™ II Taq Hot-Start DNA Polymerase (Thermo Fisher Scientific). The amplified products were purified using DNA clean and concentrator™-5 kit (Zymo Research, Irvine, CA) and sequenced in forward and reverse directions on ABI 3730 automated sequencer (Applied Biosystems Inc. Carlsbad, CA, United States).

### Statistical Analysis

Statistical significance of differences was determined by using the Student’s t test and two-way ANOVA. Results were considered statistically significant when *p* < 0.05.

## Results

### Establishment of *In Vitro* LGG Cultures

**FIGURE 1:**
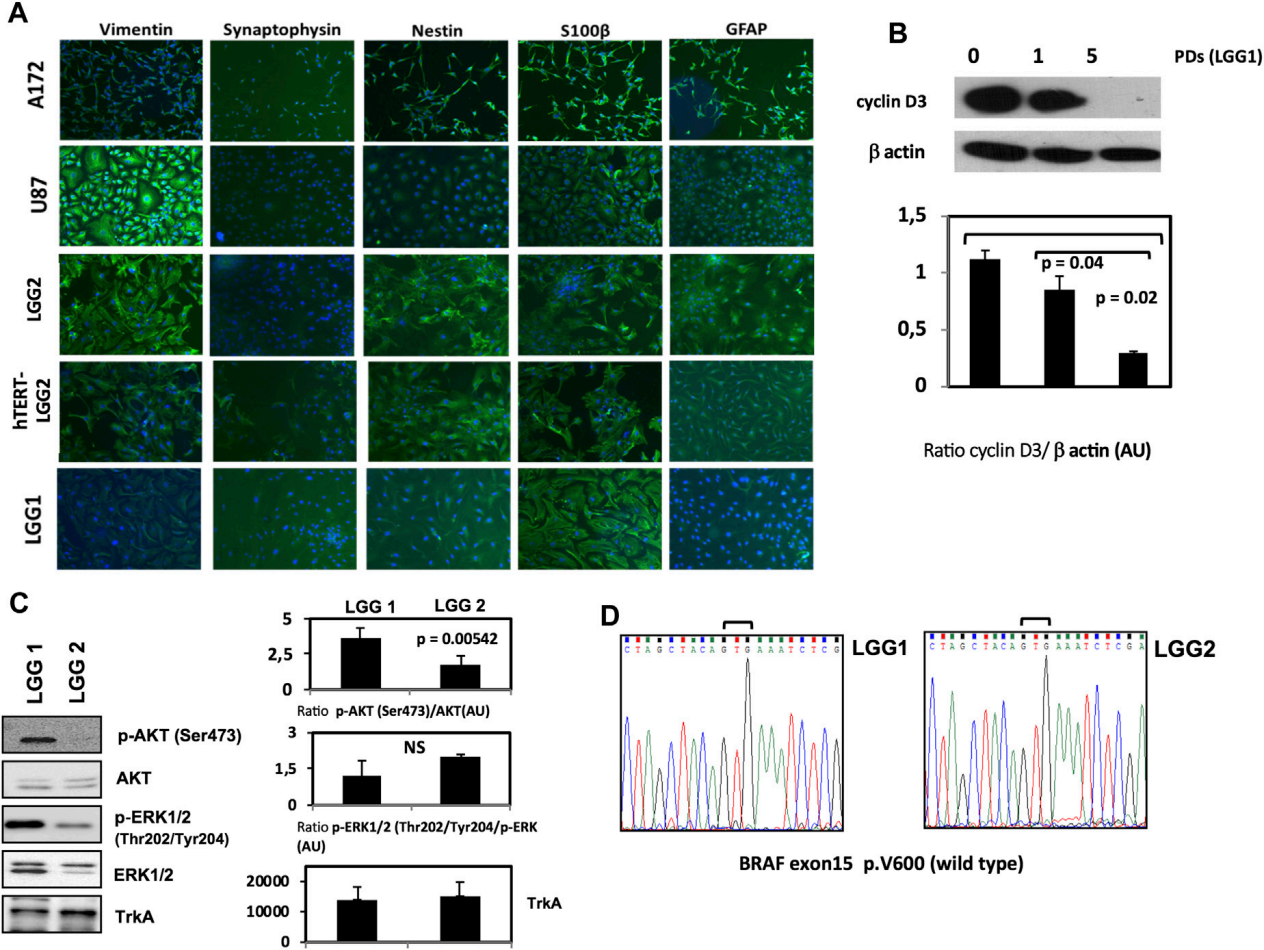
Characterization of LGG lines and generation of hTERT-LGG cells. **(A)**: Characterization of LGG cells in terms of vimentin, synaptophisin, nestin, S100 β and GFAP expression by Immunofluorescence analysis. **(B)**: representative Western Blot analysis of Cyclin D3 in pediatric low-grade astrocytoma cell line LGG1 from the start of the culture and along time culture progression. Graphs show the mean ± SD of the ratio protein/β-actin band intensity values for at least 2 independent analyses and p values were calculated using Student’s *t*-test. AU: Arbitrary Units. **(C)**: Representative Western Blot analysis of p‐AKT (Ser473), total AKT, p‐ERK1/2 (Thr202/Tyr204), total ERK1/2 and TrkA in LGG1 and LGG2 pediatric low-grade astrocytoma cell lines. Graphs show the mean ± SD of the ratio p‐AKT (Ser473)/total AKT, p‐ERK1/2 (Thr202/Tyr204)/total ERK1/2 and mean Trk A band intensity values for at least 2 independent analyses. p values were calculated using Student’s *t*-test. No significant difference was found in terms of basal p-ERK expression between LGG1 and LGG2 cells. NS: not significant. AU: Arbitrary Units. The limited number of experiments in terms of molecular characterization was due to the low amount of material provided by low proliferating LGG2 cells.**(D)**: Sequencing electropherograms of BRAF exon 15 of low grade glioma cell lines LGG1 and LGG2; no V600E mutation was identified in both LGG1 and LGG2 samples.

LGG cell lines were established *in vitro* as monolayer cultures, with doubling times higher than 72 h and a mixture of stellate and bipolar morphologies, showing cuboidal/polygonal or flattened appearance. Immunofluorescence analysis confirmed the astrocytic nature of the cells through a range of astrocyte markers such as GFAP, vimentin, S100 β, synaptophisin and nestin [38, 39] (Figure 1A).

Cyclin D3 activity is required for cell cycle and G1/S transition, and is modestly expressed in LGG cells [40]. LGG1 cells expressed cyclin D3 at the start of the culture, showing a progressive decline along the passages in culture until disappearance (Figure 1B). LGG2 cells showed similar cyclin D3 baseline expression (data not shown).

Recently, the BRAF (v-raf murine sarcoma viral oncogene homolog B) pathway, involved in the highly oncogenic RAS/RAF/MEK/ERK signaling pathway [41], has become a promising molecular target for personalized LGG therapy [42]. The most frequent mutation is a single nucleotide substitution of thymine to adenine at nucleotide 1799 that converts valine (V) to glutamic acid (E) at amino acid 600 (V600E mutation) [43].

Moreover, AKT activation has been shown to be associated with a clinically aggressive pylocitic astrocytoma phenotype [44].

Then, in order to integrate the phenotypical characterization illustrated in Figure 1, we analyzed the levels of AKT and ERK activation by Western Blot, and scrutinized the V600E BRAF mutation state of the two patient-derived LGG1 and LGG2 lines. LGG1 cells showed higher levels of AKT activation (Figure 1C), while absence of BRAF V600E mutation was observed in both LGG lines as shown by the sequencing electropherograms of Figure 1D.

**FIGURE 2:**
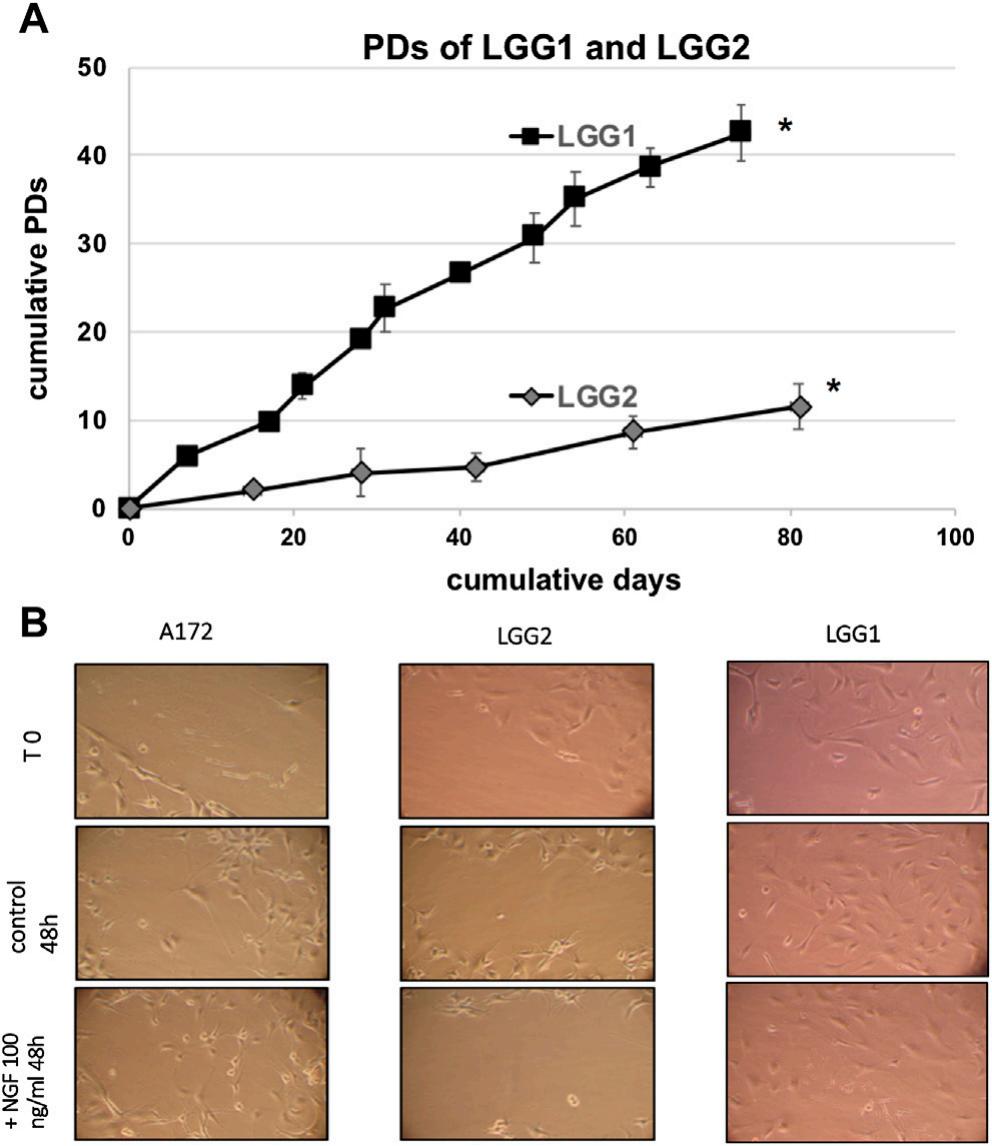
**(A)**: Proliferative capacity of two different LGG lines, named LGG1 and LGG2. Cell number was determined at the time of cell culture passages and PDs were determined as described in Material and Methods section. Graph shows the mean values obtained from at least 3 independent evaluations. Bars indicate SD. Asterisks (*) denote points after which no further expansion occurred. **(B)**: Qualitative wound closure extent was examined by “wound healing retard” assay, in which LLG cells were wounded by scratch injury induced by pipette tips in the presence of NGF. Wounds were sealed at a higher extent in LGG1 as compared with LGG2 cells at the time shown in the Figure, indicating a delay in the capacity of wound healing in LGG2. A172 cells were used as control. Images were taken at same magnification (×20).

When we analyzed the proliferative potential of the two patient-derived lines, we observed that LGG2 progressed in culture through a lower number of PDs compared to LGG1 cells (Figure 2A). PD refers to the total number of times the cells into a population have doubled since their primary isolation *in vitro*, providing an evaluation as to when the cells will reach senescence [34]. Considering the lower proliferative capacity of LGG2 line compared to LGG1, we analyzed whether this distinctive behavior was reflected in a different response to NGF treatment in terms of delay of wound healing, as a measure of cell migration and proliferative potential. The wounds induced with pipette tips in the presence of NGF were sealed at a higher extent in LGG1 as compared with LGG2 cells at the time shown in the figure (Figure 2B). These data indicate a delay in the capacity of wound healing in LGG2, providing a qualitative prediction of cell wound healing efficacy over time and suggesting a different behavior of individual LGG samples under NGF challenge.

### Generation of hTERT-LGG2 Cell Line

**FIGURE 3:**
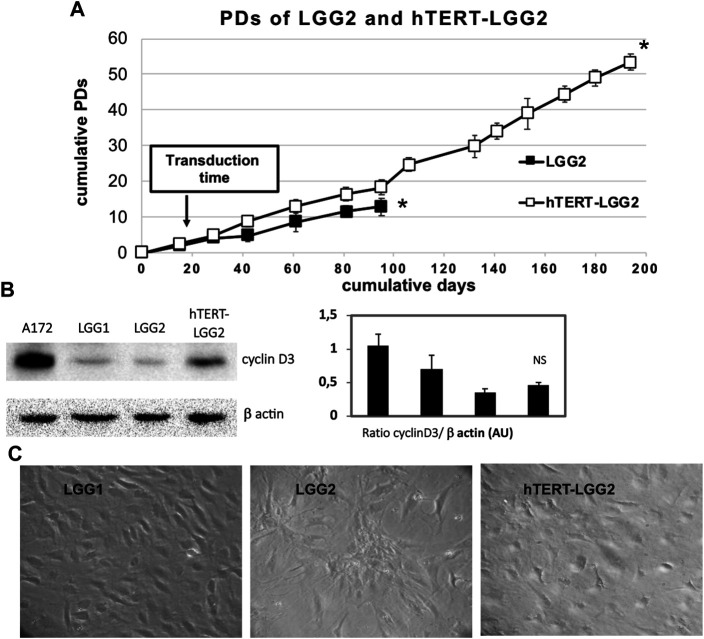
Effect of hTERT transduction on replicative capacity and senescence of LGG2. **(A)**: Proliferative capacity of hTERT-LGG2 compared to the control counterpart in terms of PDs. Cell number was determined at the time of cell culture passages and PDs were determined as described in Material and Methods section. LGG cells were grown for 6–7 PDs in vitro before they were transfected with either empty or hTERT containing vectors. Transduction with hTERT considerably extended the growth potential of low proliferating LGG2 cells. Graph shows the mean values obtained from at least 3 independent evaluations. Bars indicate SD. Asterisks (*) denote points after which no further expansion occurred. **(B)**: Cyclin D3 expression evaluated by Western Blot analysis in control LGG1 and LGG2 cell lines (10 PDs) and hTERT-LGG2 cell line (20 PDs). Graphs show the mean ± SD of the ratio protein/β-actin band intensity values for at least 2 independent analyses and p values were calculated using Student’s *t*-test. NS: not significant. AU: Arbitrary Units. The limited number of experiments in terms of molecular characterization was due to the low amount of material provided by low proliferating LGG2 cells. **(C)**: Evaluation of morphological features of LGG1, LGG2 and hTERT-LGG2 cell lines in terms of enlarged and flattened morphology, typical of senescent cells.

We investigated whether hTERT transduction could extend the proliferative potential of LGG2 cells without substantially modifying their morphological features or increasing their aggressiveness. LGG cells were grown for 6–7 PDs *in vitro* before transfection with either pHR0-CMV-hTERT-IRES2-GFP or pHR0-CMV-IRES2-GFP control lentiviral vectors. Transduction successfully produced a cell line derived from LGG2 cells, which we named hTERT-LGG2, endowed with enhanced proliferative capacity as compared with the LGG2 control cells (up to 50 PDs, Figure 3A), which reached replicative senescence rapidly, after limited rounds of duplication (Figure 3A).

**FIGURE 4:**
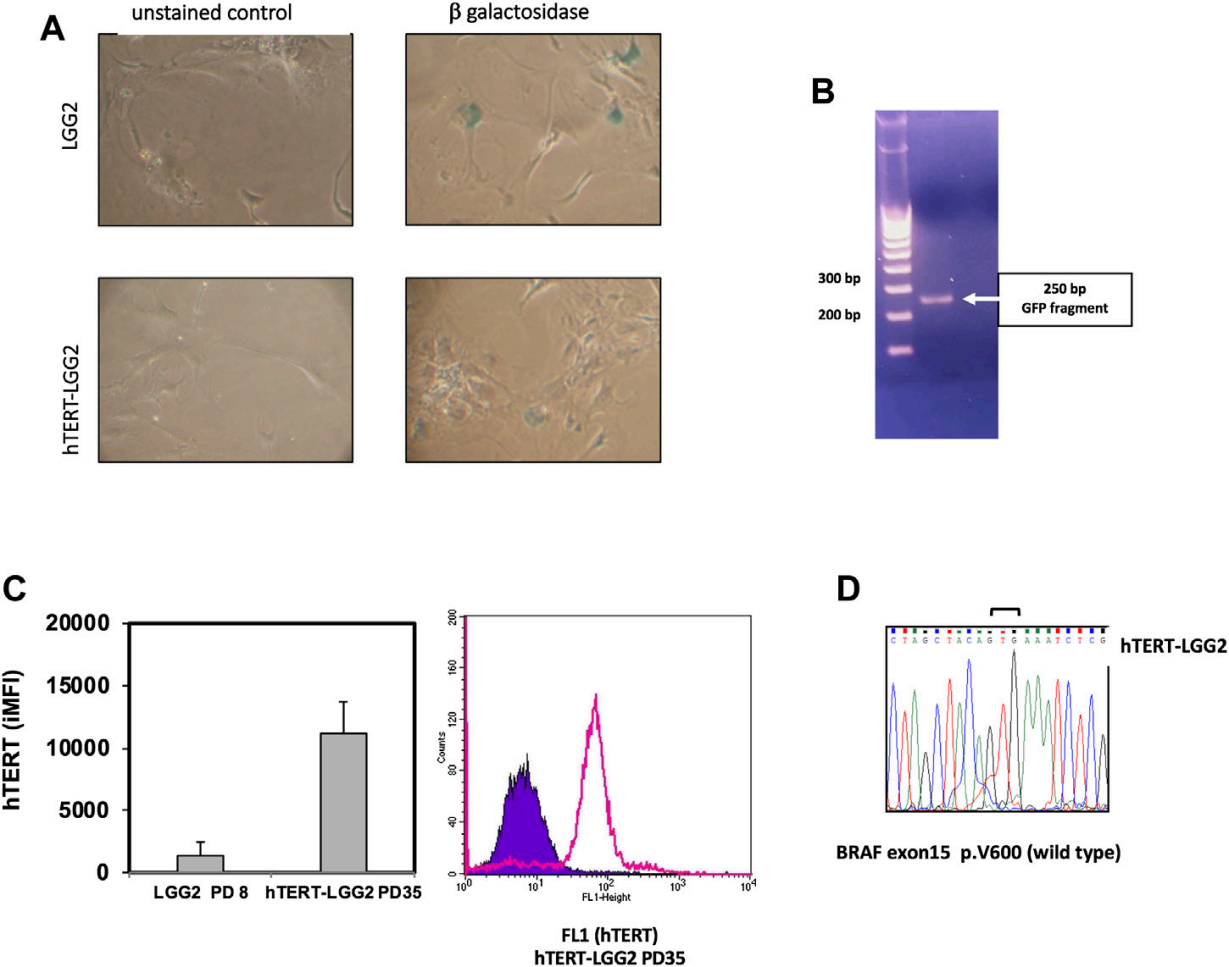
**(A)**: Estimation of β-galactosidase expression evaluated after 10°PDs (LGG2) and 20 PDs (hTERT-LGG2) cell lines, as a marker of cellular senescence. hTERT-LGG2 cell line expressed very low levels of β-galactosidase at 20 PDs, while, in contrast, LGG2 control cells exhibited high levels of the senescence marker at 10 PDs. Images were taken at same magnification (×20). **(B)**: Analysis of the integration of the GFP sequence of hTERT expressing vector into the LGG2 cell genome. PCR analysis demonstrating GFP integration (250°bp product) of genomic DNA in hTERT-LGG2 transduced cells. **(C)**: Left panel shows the histogram graph of flow-cytometric analysis of intracellular hTERT. Bars indicate the mean values of 2 independent measurements in terms of integrated MFI (iMFI). Baseline LGG2 cells showed low levels of intracellular hTERT, which were substantially increased following hTERT transduction. Bars indicate SD. A172 high grade glioma cells, used as positive control, showed off scale hTERT expression value (iMFI: 171,105, SD: 15,500). Right panel shows a representative FACS histogram plot of intracellular hTERT analysis of hTERT-LGG2 at passage 35. **(D)**: Sequencing electropherogram of BRAF exon 15 of low grade glioma cell line hTERT-LGG2; no V600E mutation was identified in hTERT-LGG2 sample.

Transduction with hTERT slightly increased cyclin D3 expression (Figure 3B). In particular, hTERT-LGG2 cells analyzed after 20 PDs showed higher cyclin D3 levels compared to those expressed by control cells evaluated after 10 PDs, when their proliferation dramatically decreased (Figure 3A,B). However, levels of cyclin D3 observed in hTERT-LGG2 cells were still much lower compared to those observed in the HGG cell line A172, used as positive control (Figure 3B). Remarkably, hTERT-LGG2 cells did not show the characteristic enlarged and flattened morphology typical of senescent cells (Figure 3C). Accordingly, while LGG2 control cells expressed high levels of β-galactosidase after 10 PDs, in correspondence of the cessation of their persistence in culture, hTERT-LGG2 expressed significantly lower levels of the senescence-related marker for at least 20 PDs (Figure 4A).

The analysis of the integration of the GFP sequence of hTERT expressing vector into the LGG2 cell genome confirmed that transduction had occurred efficiently (Figure 4B). Overexpression of hTERT in LGG2 cells was confirmed by intracellular FACS analysis, as shown by the levels of integrated Mean Fluorescence Intensity (iMFI), a measure that combines the relative frequency of molecule expression with the Mean Fluorescence Value (MFV), and by a representative FACS histogram plot showing high levels of intracellular hTERT expression after 35 PDs (Figure 4C). Absence of BRAF V600E mutation in LGG2 cells was confirmed after transduction with hTERT as shown by the sequencing electropherogram illustrated in Figure 4D.

### Effect of NGF on Proliferative, Morphological and Biochemical Features of LLG Cells

**FIGURE 5:**
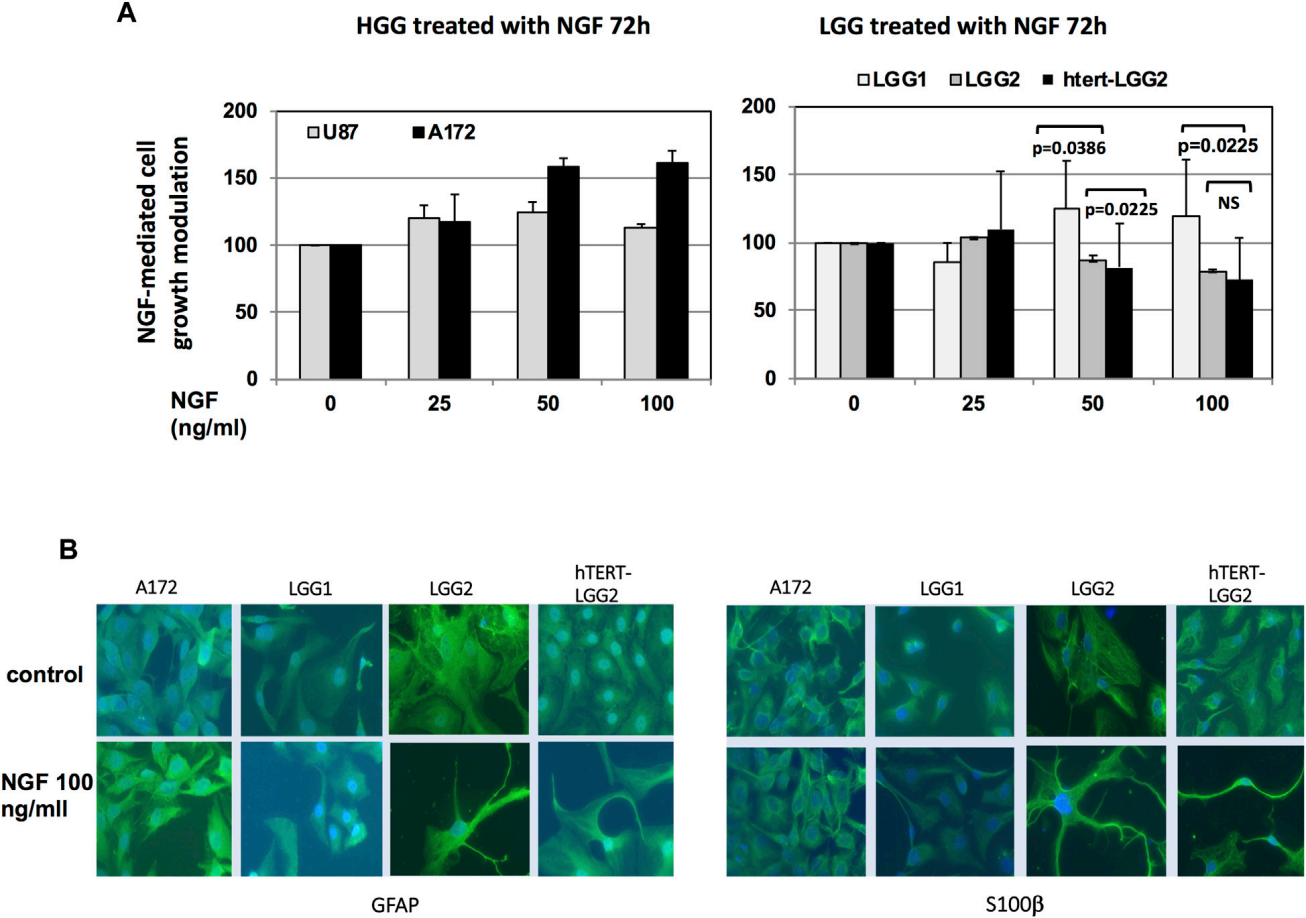
Proliferation assay and studies of cell differentiation under NGF treatment. **(A)**: Analysis of LGG cell survival following NGF treatment for 72 h in serum-free medium. Graph shows the mean of cell growth values obtained out of three independent experiments. Bars indicate SD. HGG U87 and A172 cells were used as control. Statistical analysis (two-way ANOVA test) of the differences between the effect of NGF on LGG2 and hTERT-LGG2 cell growth and on LGG1 and LGG2/hTERT-LGG2 is indicated in the graph. NS: not significant. **(B)**: Immunofluorescence analysis of NGF differentiating effects in LGG2 and h-TERT-LGG2 counterpart, as defined by cell neurite outgrowth in the presence of GFAP and S100β. Images were taken at the same magnification (×20).

Treatment with increasing concentrations of NGF produced distinctive responses in the different glioma cells analyzed, in terms of cell survival. HGG cells, used as control, showed some increase in cell growth, either with non-dose-dependent (U87) or dose-dependent behavior (A172) (Figure 5A).

Differently, LGG cells showed either little proliferation increase (LGG1) or dose-dependent growth inhibition (LGG2 and hTERT-LGG2) (Figure 5A), confirming the results obtained with the “wound healing retard” assay. No statistically significant difference was found between LGG2 and hTERT-LGG2 response to NGF in terms of cell growth, while significant divergence was observed between LGG1 and LGG2/hTERT-LGG2. Noteworthy, both hTERT-LGG2 and parental LGG2 cells exhibited growth inhibition following NGF treatment, providing a first evidence that hTERT transduction did not affect the biological response of LGG cells to NGF treatment.

We analyzed whether the potential NGF-associated differentiation resulted in the induction of neurite outgrowth in LGG cells. Immunofluorescence staining confirmed the induction of cell neurite outgrowth in the presence of GFAP astrocyte marker and differentiation indicator S100β, both in LGG2 control and h-TERT-LGG2 cells under NGF treatment (Figure 5B), demonstrating that hTERT-transduced glioma cells retained the susceptibility to NGF-mediated differentiation shown by the parental counterpart.

Then, to further analyze whether hTERT-transduced cells could provide a valid tool to study the behavior of LGG cells *in vitro*, we compared the effect of NGF in LGG2 and its hTERT-transduced counterpart on critical biochemical features involved in cell differentiation and survival.

**FIGURE 6:**
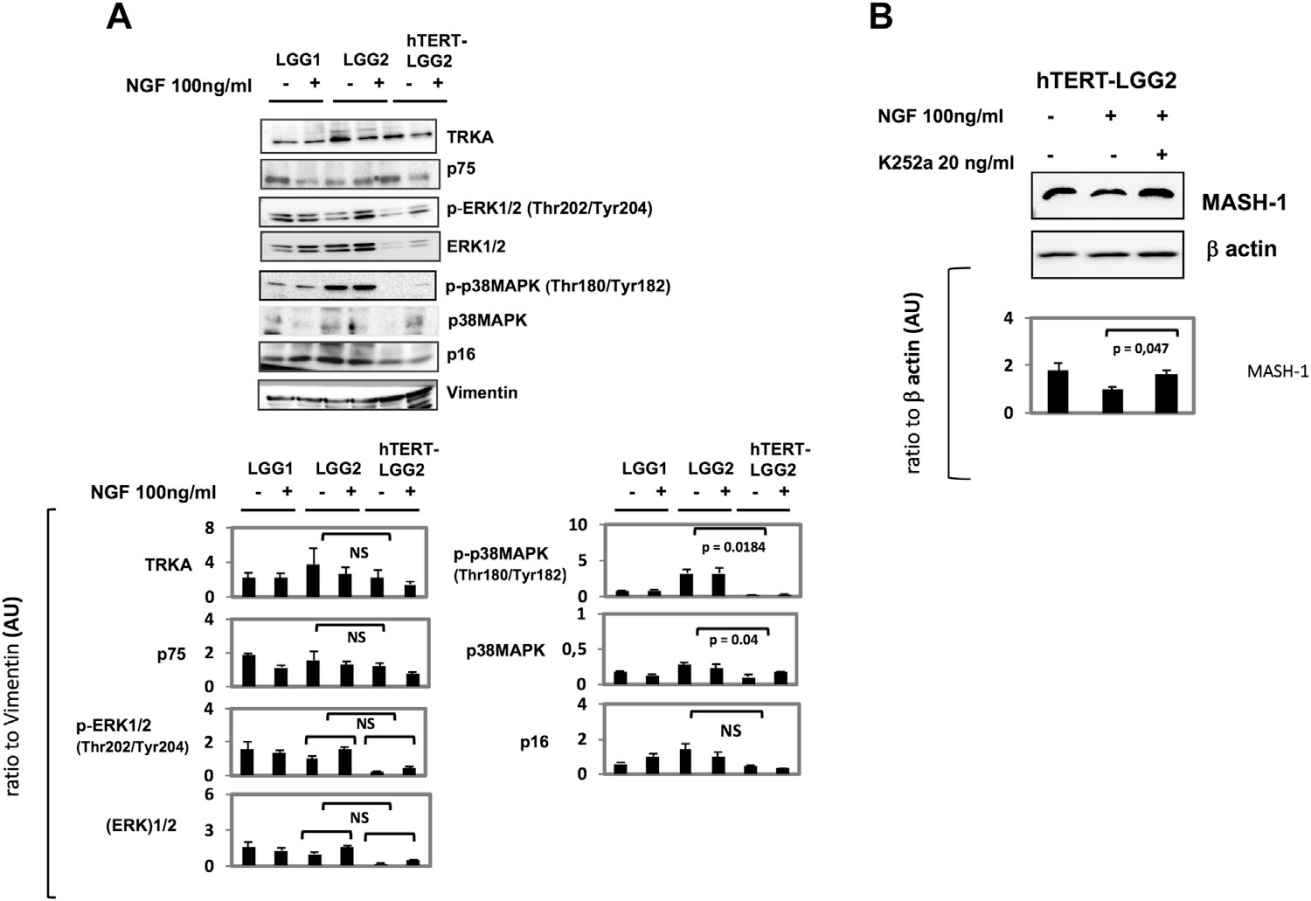
**A)**: Representative Western Blot analysis of the effect of NGF treatment on hTERT-LGG2 and the wild-type counterpart cells in terms of TrkA, p75, 44 KDa ERK, p38 MAPK and p16. Vimentin expression was tested either as astrocyte marker and loading control. pERK levels were increased by NGF in both LGG2 and hTERT transduced counterpart. P38MAPK activation and p16, both markers of cell senescence, were decreased in hTERT-LGG2 cells and not substantially modified by NGF treatment. **(B)**: Western Blot analysis of NGF-mediated modulation of MASH-1 and of the role of TrkA receptor in the NGF-induced modulation of MASH-1. hTERT-LGG2 astrocytoma cells were exposed to NGF (100 ng/ml) for 72°h in the presence or absence of the pan Trk pharmacological inhibitor K252a (20 ng/ml). MASH-1 expression was restored by K252a. Graphs show the mean ± SD of the ratio protein/Vimentin or β actin band intensity values for at least 2 independent analyses and p values were calculated using Student’s t-test. No significant difference was found in p-ERK expression between LGG2 and hTERT-LGG2 in response to NGF. NS: not significant. AU: Arbitrary Units. The limited number of experiments in terms of molecular characterization was due to the low amount of material provided by low proliferating LGG2 cells.

TrkA and p75 levels were not substantially affected by hTERT transduction (Figure 6A). Moreover, despite differences in the basal levels of ERK, both LGG2 and hTERT-LGG2 showed an increase in ERK phosphorylation. p16, evaluated as a marker of cell senescence, was decreased in hTERT-LGG2 cells, according to the strong inhibition of senescence observed in these cells, and not modified by NGF treatment both in hTERT- and control LGG2 cells (Figure 6A). Remarkably hTERT transduction strongly reduced the expression of p38 MAPK (Figure 6A).

By increasing the limited lifespan culture, transduction with hTERT provided supplementary LGG2 cells to study additional potential targets of NGF treatment. In particular, our attention focused on Achaete-scute complex homolog 1 (ASCL1 or MASH-1), a basic helix-loop-helix transcription factor essential for neuronal differentiation, which has been associated with astrocytoma progression [45, 46]. Treatment of hTERT-LGG2 cells with NGF was associated with a reduction of MASH-1 (Figure 6B). This effect was counteracted by cell-pretreatment with the pan Trk inhibitor K252a, demonstrating that TrkA receptor activation by NGF was required for MASH-1 modulation.

It should be emphasized that the goal of the present study was the increase of the proliferative capacity of low growing LGG cells in order to have a greater amount of biological material to be employed in preclinical studies of molecular characterization. It is well known the limitation of growing LGG cells, likely due to oncogene-induced senescence [20, 27]. So, the reason why we could conduct a limited amount of experiments especially in terms of molecular characterization, is that LGG2 control cells provided an insufficient amount of material to reproduce a greater number of experiments.

## Discussion

In the present study, establishment, preliminary characterization and analysis of responsivity to exogenous NGF of a patient-derived cell line and its hTERT-transduced counterpart from a human pediatric low-grade glioma is reported. The cultured cells showed anchorage-dependent growth as monolayers, with sub-populations characterized by similar morphology and cell cycle features compared to the parental control cells. Interestingly, hTERT-transduced cells showed increased PDs and molecular marker expression suggesting the achievement of increased life-span in culture without signs of conversion to high-grade glioma. Given the slow growth rate of many patient-derived low-grade primary gliomas, the difficulty to obtain cellular models for preclinical studies strongly limits the investigations on these tumors. Remarkably, hTERT alone can immortalize cells without causing cancer-associated changes or altering phenotypic properties [47–49].

Several low-grade gliomas do not proliferate adequately *in vitro*, with the consequence of not providing the adequate amount of biological material required to perform patient-specific preclinical studies. To corroborate whether hTERT-transduced LGGs can provide a reliable tool to perform preclinical studies when primary parental cells show *in vitro* poor proliferative potential, we have compared specific phenotypic and biochemical aspects between the low proliferating LGG2 and its hTERT-LGG2 counterpart as well as the proliferating primary LGG1 cell line. Importantly, LGG cell lines generated from distinct primary pediatric astrocytomas showed different proliferative potential *in vitro*, along with divergent behaviors in response to NGF treatment in terms of proliferation and wound healing delay. In particular, LGG1 cells (which proliferated spontaneously in culture for more PDs as compared with LGG2 cells), showed an increased proliferation in response to NGF, while the growth rate of LGG2 further declined after NGF treatment. Similar results were obtained in terms of proliferative and migration potential, as defined by the “wound healing retard” assay under NGF treatment. Accordingly, while both LGG1 and LGG2 showed a wild type BRAF phenotype, LGG1 exhibited higher levels of AKT activation, a condition that has been suggested to drive a more aggressive clinical phenotype [44]. This aspect can contribute explaining the different behavior of LGG1 and LGG2 in culture in terms of both PDs and response to NGF.

Remarkably, hTERT-LGG2 cells responded to NGF treatment with a behaviour comparable to that observed in the parental LGG2 cells, either in terms of cell survival/differentiation and ERK activation. hTERT transduction also strongly reduced the expression of p38 MAPK, which plays a critical role in the acceleration of cell senescence [50] and inhibition of telomerase activity [51], demonstrating a temporary escape of hTERT-LGG2 from cell senescence.

hTERT is gaining increasing attention in the molecular characterization of malignant gliomas with a different role between low and high grade. While a small subclass of adult LGG is characterized by codeletion of chromosome 1p/19q and hTERT promoter mutations [52], PAs are generally telomerase negative [29], although expressing heterogeneous levels of the catalytic telomerase subunit [30].

Considering the differential baseline behaviors and NGF responses of LGG1 and LGG2, the question arises as to what molecular characteristics are responsible for these differences and as to whether hTERT transduction would not lead to grade progression or even malignant transformation in individual tumors characterized by features different from those observed in LGG2 cell line.

Wild type BRAF genotype together with low AKT activation displayed by LGG2 cells can potentially contribute to indicate a phenotype for LGG samples whose *in vitro* survival can be increased through hTERT transduction without acquiring a more aggressive phenotype.

On the contrary, sustained and constitutive activation of AKT, the kinase responsible of intranuclear translocation and activation of hTERT [53], speaks in favor of a possible LGG grade progression following hTERT transduction.

LGG2 cells transduced with hTERT were able to grow in culture for a sufficiently long time to allow the analysis of additional biological features potentially involved in NGF-mediated glioma differentiation. NGF treatment induced a decline in the levels of MASH-1, which were restored by cell pretreatment with the pan Trk inhibitor K252a. Since a progressive increase of MASH-1 expression in grade 1 vs. grade 2–4 astrocytoma has been associated with astrocytoma prognosis [45, 46], our observations suggest a potential role of the transcription factor MASH-1 in the pro-differentiating effect of NGF through the engagement of TrkA, which can potentially contribute to antagonize astrocytoma progression [54].

**FIGURE 7:**
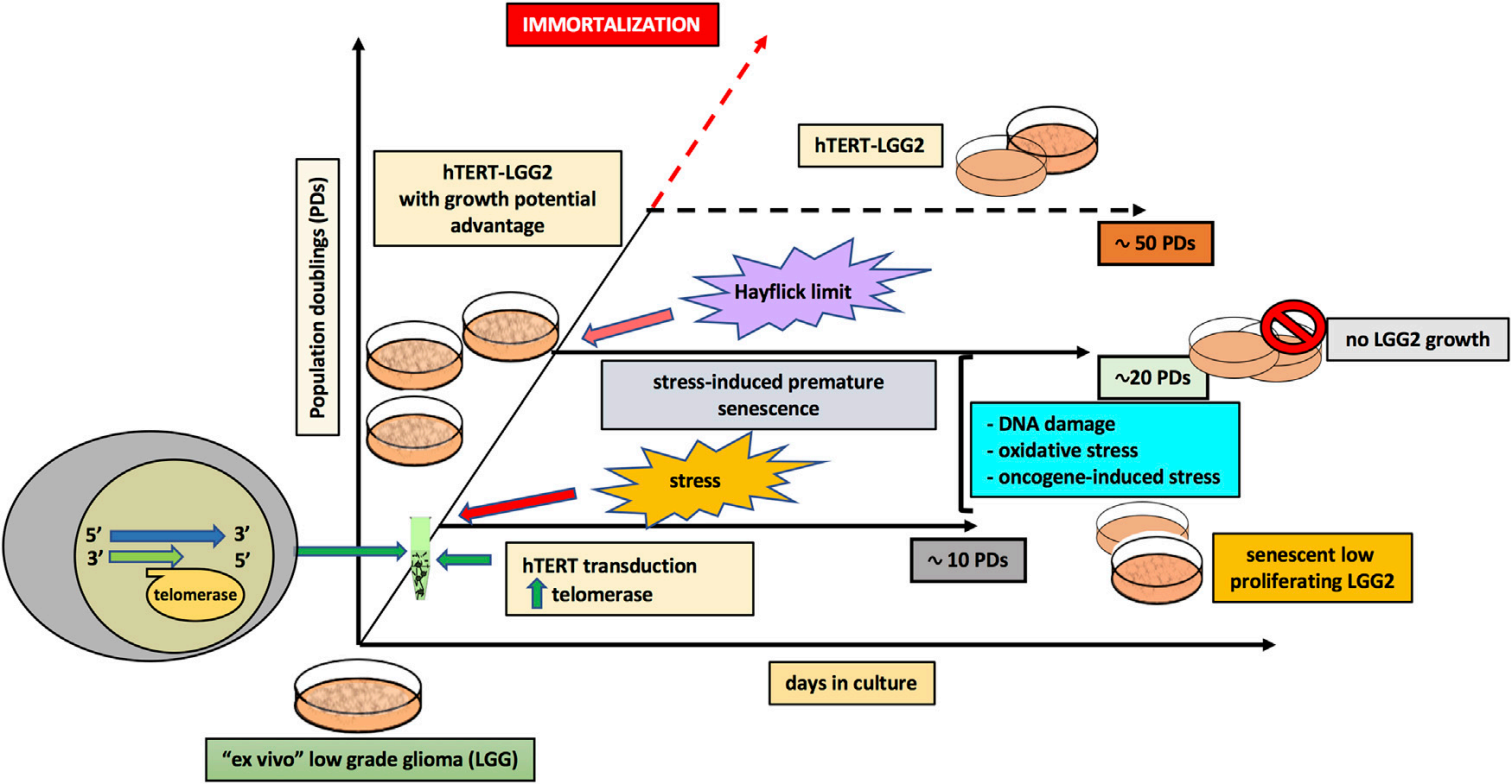
Model illustrating the role of hTERT transduction in the extended in vitro lifespan of pediatric low-grade glioma cells.

Remarkably, overexpression of telomerase in LGGs could only delay but not prevent the onset of replicative senescence, as previously observed in primary lymphocytes [34], which typically down-regulate telomerase activity upon progression into senescence [55]. In particular, hTERT transduction reasonably allowed LGG2 cells to overcome the stress-induced senescence [23, 24], which occurred after about 10 PDs in the present model, while partially counteracted the Hayflick limit due to the progressive telomere shortening as a consequence of cell proliferation [28] (Figure 7). Nevertheless, it was not sufficient to induce cellular immortalization, further supporting the absence of an increase in tumor aggressiveness by hTERT transduction.

## Conclusion

The use of personalized cell cultures can lead to the selection of the most feasible therapeutic option for LGG patients. However, this approach is not always achievable, due to the scarce proliferative potential of some patient-derived LGG cultures, which do not provide sufficient amount of biological material for deep molecular characterization and therapeutic analysis. Herein we provide preliminary lines of evidence supporting the hypothesis that hTERT-transduced LGG cells can represent a reliable experimental model for molecular and functional studies in case of selected patient-derived low-grade astrocytoma samples showing poor proliferation *in vitro*, and therefore not able to provide an adequate amount of biological material.

This approach can support the characterization of patient-specific responses and contribute to the identification of more personalized therapies.

## Data Availability

The raw data supporting the conclusions of this article will be made available by the authors, without undue reservation.

## References

[1] Qaddoumi I Sultan I Gajjar A. Outcome and prognostic features in pediatric gliomas. Cancer (2009) 115(24):5761–5770. 10.1002/cncr.24663 19813274 PMC2794938

[2] Rickert C Paulus W. Epidemiology of central nervous system tumors in childhood and adolescence based on the new WHO classification. Childs Nerv Syst (2001) 17(9):503–11. 10.1007/s003810100496 11585322

[3] Louis DN Ohgaki H Wiestler OD Cavenee WK Burger PC Jouvet A The 2007 WHO classification of tumours of the central nervous system. Acta Neuropathol (2007) 114(2):97–109. 10.1007/s00401-007-0243-4 17618441 PMC1929165

[4] Listernick R Charrow J Gutmann DH. Intracranial gliomas in neurofibromatosis type 1. Am J Med Genet (1999) 89:38–44. 10.1002/(sici)1096-8628(19990326)89:1<38::aid-ajmg8>3.0.co;2-m 10469435

[5] Stokland T Liu J-F Ironside JW Ellison DW Taylor R Robinson KJ A multivariate analysis of factors determining tumor progression in childhood low-grade glioma: a population-based cohort study (CCLG CNS9702). Neuro Oncol (2010) 12(12):1257–68. 10.1093/neuonc/noq092 20861086 PMC3018938

[6] Falsini B Chiaretti A Rizzo D Piccardi M Ruggiero A Manni L Nerve growth factor improves visual loss in childhood optic gliomas: a randomized, double-blind, phase II clinical trial. Brain (2016) 139(2):404–14. 10.1093/brain/awv366 26767384

[7] Falsini B Chiaretti A Barone G Piccardi M Pierri F Colosimo C Topical nerve growth factor as a visual rescue strategy in pediatric optic gliomas. Neurorehabil Neural Repair (2011) 25(6):512–20. 10.1177/1545968310397201 21444653

[8] Chao MV. Nerve growth factor. In: Sporn MB Roberts AB. , editors. Peptide growth factors and their receptors II. New York, NY: Springer Publishing Co (1991). 135–65.

[9] Frade JM Barde Y-A. Nerve growth factor: two receptors, multiple functions. Bioessays (1998) 20(2):137–45. 10.1002/(SICI)1521-1878(199802)20:2<137::AID-BIES6>3.0.CO;2-Q 9631659

[10] Chiaretti A Falsini B Aloe L Pierri F Fantacci C Riccardi R. Neuroprotective role of nerve growth factor in hypoxicischemic injury. From brain to skin. Arch Ital Biol (2011) 149(2):275–82. 10.4449/aib.v149i2.1364 21702000

[11] Liu TT Wang H Wang FJ Xi YF Chen LH. Expression of nerve growth factor and brain-derived neurotrophic factor in astrocytomas. Oncol Lett (2018) 15(1):533–7. 10.3892/ol.2017.7333 29391888 PMC5769415

[12] Meco D Di Francesco AM Melotti L Ruggiero A Riccardi R. Ectopic nerve growth factor prevents proliferation in glioma cells by senescence induction. J Cell Physiol (2019) 234(5):6820–30. 10.1002/jcp.27430 30417351

[13] Segal RA Greenberg ME. Intracellular signaling pathways activated by neuropathic factors. Annu Rev Neuro Sci (1996) 19:463–89. 10.1146/annurev.ne.19.030196.002335 8833451

[14] Kraemer BR Snow JP Vollbrecht P Pathak A Valentine WM Deutch AY A role for the p75 neurotrophin receptor in axonal degeneration and apoptosis induced by oxidative stress. J Biol Chem (2014) 289(31):21205–16. 10.1074/jbc.M114.563403 24939843 PMC4118083

[15] Cragnolini AB Huang Y Gokina P Friedman WJ. Nerve growth factor attenuates proliferation of astrocytes via the p75 neurotrophin receptor. Glia (2009) 57(13):1386–92. 10.1002/glia.20857 19229990 PMC2735589

[16] Aloe L Alleva E Böhm A Levi-Montalcini R. Aggressive behavior induces release of nerve growth factor from mouse salivary gland into the bloodstream. Proc Natl Acad Sci U S A (1986) 83(16):6184–7. 10.1073/pnas.83.16.6184 3090553 PMC386464

[17] Kimura S Yoshino A Katayama Y Watanabe T Fukushima T. Growth control of C6 glioma *in vivo* by nerve growth factor. J Neurooncol (2002) 59(3):199–205. 10.1023/a:1019919019497 12241115

[18] Brown MC Staniszewska I Lazarovici P Tuszynski GP Del Valle L Marcinkiewicz C. Regulatory effect of nerve growth factor in α9β1 integrin-dependent progression of glioblastoma. Neuro Oncol (2008) 10(6):968–80. 10.1215/15228517-2008-04710.1215/15228517-2008-0047 19074980 PMC2719011

[19] Tada K Kochi M Saya H Kuratsu J-i. Shiraishi S Kamiryo T Preliminary observations on genetic alterations in pilocytic astrocytomas associated with neurofibromatosis 1. Neuro Oncol (2003) 5(4):228–34. 10.1215/S115285170300005X 14565158 PMC1920681

[20] Jacob K Quang-Khuong D-A Jones DTW Witt H Lambert S Albrecht S Genetic aberrations leading to MAPK pathway activation mediate oncogene-induced senescence in sporadic pilocytic astrocytomas. Clin Cancer Res (2011) 17(14):4650–60. 10.1158/1078-0432.CCR-11-0127 21610151

[21] Descamps S Toillon R-A Adriaenssens E Pawlowski V Cool SM Nurcombe V Nerve growth factor stimulates proliferation and survival of human breast cancer cells through two distinct signaling pathways. J Biol Chem (2001) 276(21):17864–17870. 10.1074/jbc.M010499200 11359788

[22] Stoczynska-Fidelus E Och W Rieske P Bienkowski M Banaszczyk M Winiecka-Klimek M Spontaneous *in vitro* senescence of glioma cells confirmed by an antibody against IDH1R132H. Anticancer Res (2014) 34(6):2859–67. 24922649

[23] Courtois-Cox S Genther Williams SM Reczek EE Johnson BW McGillicuddy LT Johannessen CM A negative feedback signaling network underlies oncogene-induced senescence. Cancer Cell (2006) 10(6):459–72. 10.1016/j.ccr.2006.10.003 17157787 PMC2692661

[24] Sarkisian CJ Keister BA Stairs DB Boxer RB Moody SE Chodosh LA. Dose-dependent oncogene-induced senescence *in vivo* and its evasion during mammary tumorigenesis. Nat Cell Biol (2007) 9(5):493–505. 10.1038/ncb1567 17450133

[25] Broniscer A Baker SJ West AN Fraser MM Proko E Kocak M Clinical and molecular characteristics of malignant transformation of low-grade glioma in children. J Clin Oncol (2007) 25(6):682–9. 10.1200/JCO.2006.06.8213 17308273

[26] Cheng Y Pang JC-S Ng H-K Ding M Zhang S-F Zheng J Pilocytic astrocytomas do not show most of the genetic changes commonly seen in diffuse astrocytomas. Histopathology (2000) 37(5):437–44. 10.1046/j.1365-2559.2000.01005.x 11119125

[27] Lin AW Barradas M Stone JC van Aelst L Serrano M Lowe SW. Premature senescence involving p53 and p16 is activated in response to constitutive MEK/MAPK mitogenic signaling. Genes Dev (1998) 12(19):3008–19. 10.1101/gad.12.19.3008 9765203 PMC317198

[28] Harley CB. Telomerase is not an oncogene. Oncogene (2002) 21(4):494–502. 10.1038/sj.onc.1205076 11850774

[29] Chong EY Lam PY Poon W-S Ho-Kueng NG. Telomerase expression in gliomas including the nonastrocytic tumors. Hum Pathol (1998) 29(6):599–603. 10.1016/s0046-8177(98)80009-9 9635680

[30] Kotoula V Cheva A Barbanis S Papadimitriou CS Karkavelas G. hTERT immunopositivity patterns in the normal brain and in astrocytic tumors. Acta Neuropathol (2006) 111:569–78. 10.1007/s00401-006-0036-1 16614861

[31] Maes L Van Neste L Van Damme K Kalala J De Ridder L Bekaert S Relation between telomerase activity, hTERT and telomere length for intracranial tumours. Oncol Rep (2007) 18(6):1571–6. 10.3892/or.18.6.1571 17982646

[32] Maes L Kalala JPO Cornelissen M de Ridder L. Progression of astrocytomas and meningiomas: an evaluation *in vitro* . Cell Prolif (2007) 40(1):14–23. 10.1111/j.1365-2184.2007.00415.x 17227292 PMC6496744

[33] Sonoda Y Ozawa T Hirose Y Aldape KD McMahon M Berger MS Formation of intracranial tumors by genetically modified human astrocytes defines four pathways critical in the development of human anaplastic astrocytoma. Cancer Res (2001) 61(13):4956–60. 11431323

[34] Plunkett FJ Franzese O Belaramani LL Fletcher JM Gilmour KC Sharifi R The impact of telomere erosion on memory CD8+ T cells in patients with X-linked lymphoproliferative syndrome. Mech Ageing Dev (2005) 126(8):855–65. 10.1016/j.mad.2005.03.006 15992610

[35] Forte G Franzese O Pagliari S Pagliari F Di Francesco AM Cossa P Interfacing sca-1posMesenchymal stem cells with biocompatible scaffolds with different chemical composition and geometry. J Biomed Biotechnol (2009) 2009:910610. 10.1155/2009/910610 19644551 PMC2715823

[36] Mizuguchi H Xu Z Ishii-Watabe A Uchida E Hayakawa T. IRES-dependent second gene expression is significantly lower than cap-dependent first gene expression in a bicistronic vector. Mol Ther (2000) 1(4):376–82. 10.1006/mthe.2000.0050 10933956

[37] Berdún S Rychter J Vergara P. Effects of nerve growth factor antagonist K252a on peritoneal mast cell degranulation: implications for rat postoperative ileus. Am J Physiol Gastrointest Liver Physiol (2015) 309(10):G801–G806. 10.1152/ajpgi.00152.2015 26405114

[38] Ito K Sanosaka T Igarashi K Ideta-OtsukaIdeta-Otsuka M Aizawa A Uosaki Y Identification of genes associated with the astrocyte-specific gene Gfap during astrocyte differentiation. Sci Rep (2016) 6:23903. 10.1038/srep23903 27041678 PMC4819225

[39] Bax DA Little SE Gaspar N Perryman L Marshall L Viana-Pereira M Molecular and phenotypic characterisation of paediatric glioma cell lines as models for preclinical drug development. PLoS One (2009) 4(4):e5209. 10.1371/journal.pone.0005209 19365568 PMC2666263

[40] Zhang X Zhao M Huang A-y. Fei Z Zhang W Wang X-l. The effect of cyclin D expression on cell proliferation in human gliomas. J Clin Neurosci (2005) 12(2):166–8. 10.1016/j.jocn.2004.03.036 15749420

[41] Lavoie H Therrien M. Regulation of RAF protein kinases in ERK signalling. Nat Rev Mol Cell Biol (2015) 16(5):281–98. 10.1038/nrm3979 25907612

[42] Behling F Schittenhelm J. Oncogenic BRAF alterations and their role in brain tumors. Cancers (Basel) (2019) 11(6):794. 10.3390/cancers11060794 PMC6627484 31181803

[43] Davies H Bignell GR Cox C Stephens P Edkins S Clegg S Mutations of the BRAF gene in human cancer. Nature (2002) 417(6892):949–954. 10.1038/nature00766 12068308

[44] Rodriguez EF Scheithauer BW Giannini C Rynearson A Cen L Hoesley B PI3K/AKT pathway alterations are associated with clinically aggressive and histologically anaplastic subsets of pilocytic astrocytoma. Acta Neuropathol (2011) 121(3):407–420. 10.1007/s00401-010-0784-9 21113787 PMC3417062

[45] Kim EJ Battiste J Nakagawa Y Johnson JE. Ascl1 (Mash1) lineage cells contribute to discrete cell populations in CNS architecture. Mol Cell Neurosci (2008) 38(4):595–606. 10.1016/j.mcn.2008.05.008 18585058 PMC2570020

[46] Somasundaram K Reddy SP Vinnakota K Britto R Subbarayan M Nambiar S Upregulation of ASCL1 and inhibition of Notch signaling pathway characterize progressive astrocytoma. Oncogene (2005) 24(47):7073–83. 10.1038/sj.onc.1208865 16103883

[47] Weng NP Hodes RJ. The role of telomerase expression and telomere length maintenance in human and mouse. J Clin Immunol (2000) 20(4):257–67. 10.1023/a:1017223602293 10939713

[48] Lee KM Choi KH Ouellette MM. Use of exogenous hTERT to immortalize primary human cells. Cytotechnology (2004) 45(1-2):33–8. 10.1007/10.1007/s10616-004-5123-3 19003241 PMC3449956

[49] Tentori L Vergati M Muzi A Levati L Ruffini F Forini O Generation of an immortalized human endothelial cell line as a model of neovascular proliferating endothelial cells to assess chemosensitivity to anticancer drugs. Int J Oncol (2005) 27(2):525–35. 10.3892/ijo.27.2.525 16010436

[50] Davis T Brook A Rokicki MJ Bagley MC Kipling D. Evaluating the role of p38 MAPK in the accelerated cell senescence of werner syndrome fibroblasts. J Pharm (2016) 9(2):23. 10.3390/ph9020023 PMC4932541 27136566

[51] Lanna A Coutavas E Levati L Seidel J Rustin MH Henson SM IFN-α inhibits telomerase in human CD8⁺ T cells by both hTERT downregulation and induction of p38 MAPK signalling. J Immunol (2013) 191(7):3744–52. 10.4049/jimmunol.1301409 23997212 PMC3836248

[52] Louis DN Perry A Reifenberger G von Deimling A Figarella-Branger D Cavenee WK The 2016 world health organization classification of tumors of the central nervous system: a summary. Acta Neuropathol (2016) 131(6):803–20. 10.1007/s00401-016-1545-1 27157931

[53] Comandini A Naro C Adamo R Akbar AN Lanna A Bonmassar E Molecular mechanisms involved in HIV-1-tat mediated inhibition of telomerase activity in human CD4+ T lymphocytes. Mol Immunol (2013) 54(2):181–92. 10.1016/j.molimm.2012.12.003 23287597

[54] Tacha DE Tyrrell J Lobo M. MASH-1 expression in high grade astrocytoma may demonstrate a potential model for prognosis that may lead to a molecular target for future therapy. In: Proceedings of the 107th Annual Meeting of the American Association for Cancer Research; 2016 April 16–20; New Orleans, LA [abstract] (2016).

[55] Franzese O Barbaccia ML Bonmassar E Graziani G. Beneficial and detrimental effects of antiretroviral therapy on HIV-associated immunosenescence. Chemotherapy (2018) 63(2):64–75. 10.1159/000487534 29533947

